# Expression of human protein S100A7 (psoriasin), preparation of antibody and application to human larynx squamous cell carcinoma

**DOI:** 10.1186/1756-0500-4-494

**Published:** 2011-11-14

**Authors:** Manuela R Barbieri, Camillo DC Andrade, Wilson A Silva, Adriana A Marques, Andréia M Leopoldino, Marlise BA Montes, Marcelo Dias-Baruffi, Iberê C Soares, Alda Wakamatsu, Venâncio AF Alves, Hélen J Laure, Marco A Zago, Lewis J Greene

**Affiliations:** 1 Department of Clinical Medicine, Faculty of Medicine of Ribeirão Preto, University of São Paulo, Ribeirão Preto, SP, Brazil; 2 Department of Genetics, Faculty of Medicine of Ribeirão Preto, University of São Paulo, Ribeirão Preto, SP, Brazil; 3 Department of Clinical, Toxicological and Bromatological Analysis, Faculty of Pharmaceutical Sciences of Ribeirão Preto, University of São Paulo, Ribeirão Preto, SP, Brazil; 4 Department of Pathology, Faculty of Medicine of São Paulo, University of São Paulo, São Paulo, SP, Brazil; 5 Department of Molecular and Cellular Biology and Pathogenic Bioagents, Faculty of Medicine of Ribeirão Preto, University of São Paulo, Ribeirão Preto, SP, Brazil; 6 Center for Cellular Therapy and Hemotherapy of Ribeirão Preto, Faculty of Medicine of Ribeirão Preto, University of São Paulo, Ribeirão Preto, SP, Brazil; 7 Laboratory of Molecular Genetics, Faculty of Medicine of Ribeirão Preto, University of São Paulo, Ribeirão Preto, SP, Brazil; 8 Protein Chemistry Center, Faculty of Medicine of Ribeirão Preto, University of São Paulo, Ribeirão Preto, SP, Brazil; 9 Hemotherapy Regional Center, Center for Protein Chemistry, Tenente Catão Roxo, 2501, Monte Alegre, 14049-900, Ribeirão Preto, SP, Brazil

**Keywords:** S100A7 (Psoriasin), Recombinant protein, Production of a polyclonal antibody, *E. coli *BL21::DE3, Mass spectrometry

## Abstract

**Background:**

Up-regulation of S100A7 (Psoriasin), a small calcium-binding protein, is associated with the development of several types of carcinomas, but its function and possibility to serve as a diagnostic or prognostic marker have not been fully defined. In order to prepare antibodies to the protein for immunohistochemical studies we produced the recombinant S100A7 protein in *E. coli*. mRNA extracted from human tracheal tumor tissue which was amplified by RT-PCR to provide the region coding for the S100A7 gene. The amplified fragment was cloned in the vector pCR2.1-TOPO and sub-cloned in the expression vector pAE. The protein rS100A7 (His-tag) was expressed in *E. coli *BL21::DE3, purified by affinity chromatography on an Ni-NTA column, recovered in the 2.0 to 3.5 mg/mL range in culture medium, and used to produce a rabbit polyclonal antibody anti-rS100A7 protein. The profile of this polyclonal antibody was evaluated in a tissue microarray.

**Results:**

The rS100A7 (His-tag) protein was homogeneous by SDS-PAGE and mass spectrometry and was used to produce an anti-recombinant S100A7 (His-tag) rabbit serum (polyclonal antibody anti-rS100A7). The molecular weight of rS100A7 (His-tag) protein determined by linear MALDI-TOF-MS was 12,655.91 Da. The theoretical mass calculated for the nonapeptide attached to the amino terminus is 12,653.26 Da (delta 2.65 Da). Immunostaining with the polyclonal anti-rS100A7 protein generated showed reactivity with little or no background staining in head and neck squamous cell carcinoma cells, detecting S100A7 both in nucleus and cytoplasm. Lower levels of S100A7 were detected in non-neoplastic tissue.

**Conclusions:**

The polyclonal anti-rS100A7 antibody generated here yielded a good signal-to-noise contrast and should be useful for immunohistochemical detection of S100A7 protein. Its potential use for other epithelial lesions besides human larynx squamous cell carcinoma and non-neoplastic larynx should be explored in future.

## Background

Head and neck squamous cell carcinomas (HNSCC) are among the most common types of neoplasias and their relative incidence has increased recently due to the rising life expectancy of the population [1]. HNSCC are tumors of epithelial origin which can involve the oral cavity, pharynx and larynx. Their main risk factors are exposure to alcohol and tobacco [2]. Changes triggered in genes involved in the regulation of important cell functions may result in disordered proliferation and the invasion of other tissues [3]. As is the case for most neoplasias, there are no specific biomarkers for HNSCC. In a study of the serial analysis gene expression (SAGE) of human larynx tumor tissue several differentially expressed genes were identified, among them, the up-regulation of the S100A7 gene [4] belonging to a family of calcium-binding proteins. S100A7 has been considered to be a marker for tumor progression in oral neoplasias [5], as well as in breast [6] and ovarian cancer [7] on the basis of immunohistochemistry; however, its expression in normal tissue appears to preclude its use as a specific cancer marker [8].

The S100A7 protein, also called psoriasin, was detected in keratinocytes of psoriatic epidermis. The S100A7 gene is organized in 3 exons and consists of 306 base pairs, and the translated protein has 101 amino acids and a molecular mass of 11,457 Da calculated on the basis of amino acid composition [9]. The protein S100A7 is a member of the S100 protein family containing two calcium-binding domains denoted "2EF hand". Thus, the protein contains a calcium-binding domain in the N-terminal region which includes an additional three amino acids when compared to the other S100 proteins, and a binding domain in the C-terminal region containing the structural motif "EF hand" [10]. S100 proteins have been implicated in a variety of intracellular and extracellular functions and are involved in regulation of protein phosphorylation, transcription factors, Ca^2+ ^homeostasis, the dynamics of cytoskeleton constituents, enzyme activities, cell growth and differentiation, and the inflammatory response. The S100 protein family contains 21 members [11] that are located within the S100 gene cluster in the q21 region of chromosome 1 [12].

Because several lines of evidence suggested that S100A7 might be a biomarker for tumor progression, we prepared the recombinant protein and polyclonal antibodies to it. At that time we were not aware of commercially available antibodies.

In the present study, the rS100A7 (His-tag) protein was expressed in *E. coli *BL21::DE3, purified by affinity chromatography on an Ni-NTA column and characterized by SDS-PAGE, Western blot and mass spectrometry. The homogeneous rS100A7 (His-tag) protein was used to produce the polyclonal antibody anti-rS100A7 which was applied to microarrays of tissues containing human larynx squamous cell carcinoma and non-neoplastic tissues.

## Results and discussion

### Amplification and cloning of the S100A7 gene

**Figure 1:**
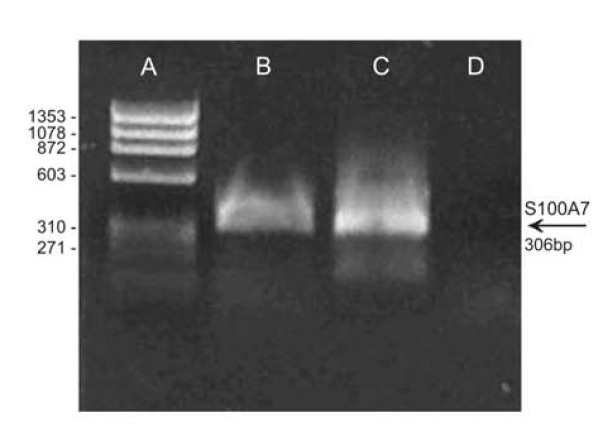
**Electrophoretic profile of the DNA fragment corresponding to the S100A7 gene after PCR amplification on 1.5% agarose gel (w/v)**. **a**, θX174 DNA ladder in kbp; **b**, amplification of cDNA of tracheal head and neck squamous cell carcinoma (HNSCC) tissue; **c**, nested PCR of amplification of the DNA fragment, with the presence of the fragment of approximately 306 bp in both lanes; **d**, control (PCR without template).

Amplification of the S100A7 gene by PCR (Figure 1a) and nested PCR (Figure 1b) was confirmed in 1.0% agarose gel (w/v) electrophoresis by detection of the expected 306 bp fragment (Figure 1). The fragment corresponding to gene S100A7 was sub-cloned in an expression vector (pAE/S100A7) after cleavage with the restriction enzymes *Xho*I and *Hind*III. The correct insertion, identity and integrity of gene S100A7 were demonstrated on the basis of DNA sequencing which presented an identical sequence to that in GenBank Accession number 002963 (data not shown). The recombinant plasmid (pAE/S100A7) was then introduced into *E. coli *BL21::DE3 for the expression of recombinant S100A7 (His-tag) protein.

### Expression and purification of the recombinant S100A7 (His-tag) protein

**Figure 2:**
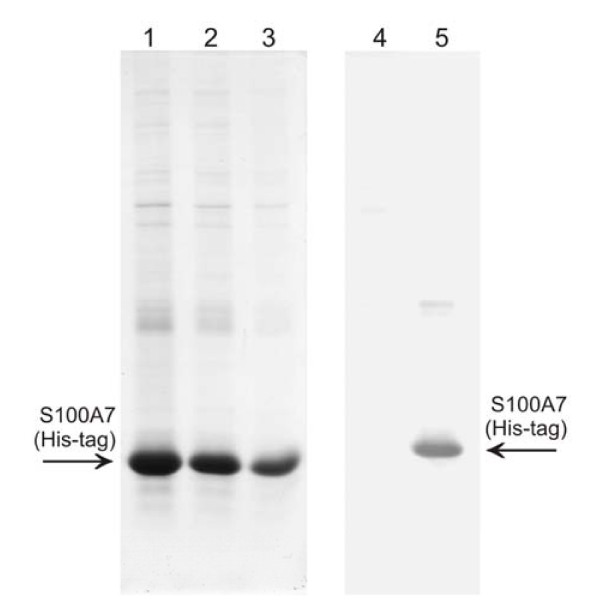
**Expression and homogeneity of rS100A7 (His-tag) protein**. SDS-PAGE 12.5% electrophoresis of rS100A7 (His-tag) protein. The protein was expressed in *E. coli *BL21::DE3 and purified on an Ni-NTA affinity column. Lanes 1, 2 and 3, are the 1st, 2nd and 3 rd effluents of the column washed with 8 M urea. The gel was stained with Coomassie blue G. Lane 4 is an extract of *E. coli *which were not transformed (negative control). Lane 5 is a western blot using anti-recombinant S100A7 (His-tag) rabbit serum at 1:5000 dilution. The arrows indicate the electrophoretic position corresponding to rS100A7 (His-tag) protein.

The expression of rS100A7 (His-tag) protein fused with His-tag was carried out in *E. coli *BL21DE3. The highest amount of recombinant proteins was obtained when the IPTG inducer was not added to the culture medium. The rS100A7 (His-tag) protein was present in inclusion bodies which were denatured in 8 M urea before purification with the Ni-NTA affinity column under denaturing conditions. The yield of rS100A7 (His-tag) protein was 2.0 to 3.5 mg/mL culture medium. Purified rS100A7 (His-tag) protein (200 ng) was homogeneous by 12.5% SDS-PAGE and Western blotting using the anti-S100A7 (His-tag) polyclonal antibody described here (Figure 2).

### Identification of rS100A7 (His-tag) protein by mass spectrometry

**Figure 3:**
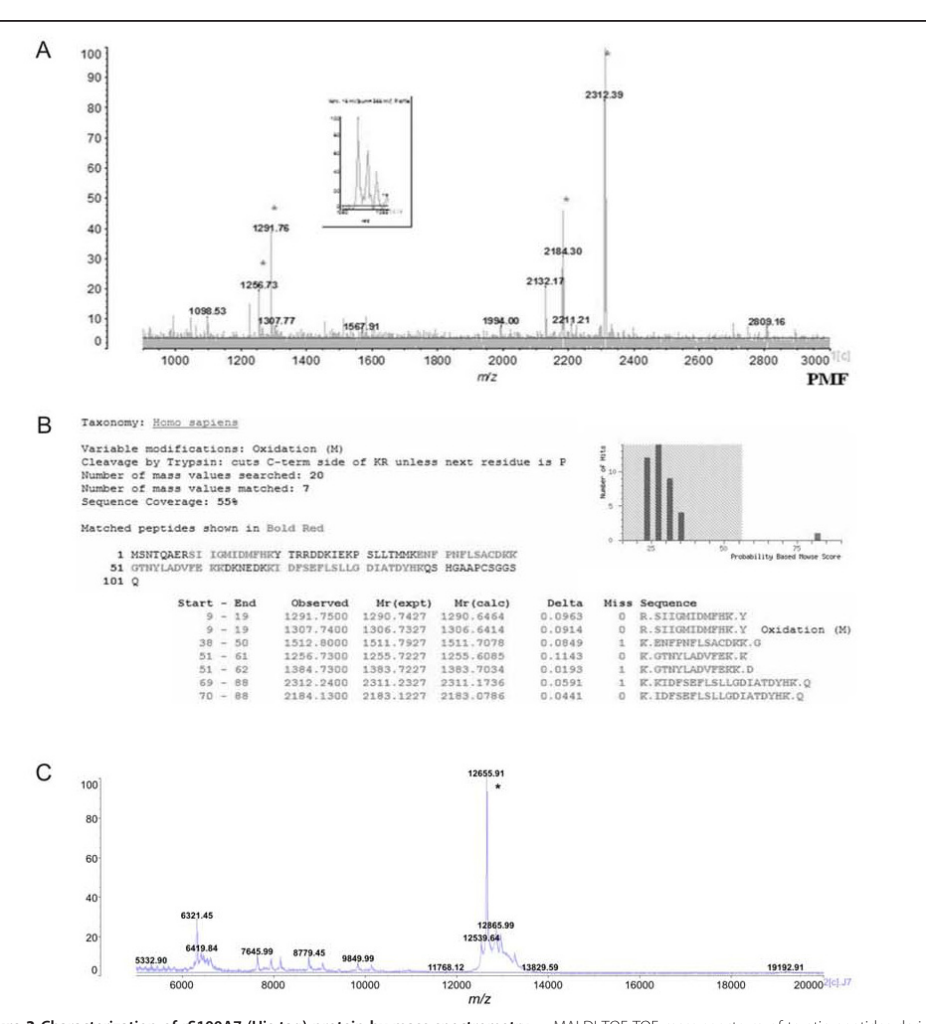
**Characterization of rS100A7 (His-tag) protein by mass spectrometry**. **a**, MALDI-TOF-TOF mass spectrum of tryptic peptides derived from rS100A7 (His-tag) protein. **b**, Mascot data obtained by analysis of the tryptic peptides shown in panel B. **c**, MALDI-TOF-TOF mass spectrometric determination of the molecular mass of intact rS100A7 (His-tag) protein.

We did not try to remove the nonapeptide from rS100A7 (His-tag). The rS100A7 (His-Tag) protein was purified by SDS-PAGE, treated with trypsin and the hydrolysate analyzed by MALDI-TOF-MS (Figure 3a). Ions with the highest intensity were sequenced by CID-MS/MS. The peptides identified by sequencing accounted for 55% of the amino acid sequence of the S100A7 protein acquisition number P31151, Swiss-Prot data base (Figure 3b).

The protein rS100A7 (His-Tag) described here has a nonapeptide, MHHHHHHLE, attached to its amino terminus. The molecular mass of the intact protein determined by linear MALDI-TOF-MS (Figure 3c) was 12,655.91 Da compared to a calculated molecular mass of 12,653.26 Da (delta = + 2.65 Da) (ExPasy Proteomics Server, http://expasy.org). We never had the rS100A7 protein without the His-tag.

### Western blot analysis of rS100A7 (His-Tag)

The gel in Figure 2, lane 1, shows that only a primary component in the purified recombinant protein was detected by the antibody or by Coomassie Blue staining and that the mobility in SDS-PAGE corresponded to a mass of approximately 11.5 kDa. A second component in much lower concentration is visible at a higher molecular weight and may be an oligomer.

### Immunohistochemical analysis of the anti-S100A7 (His-tag) polyclonal antibody using TMA

**Figure 4:**
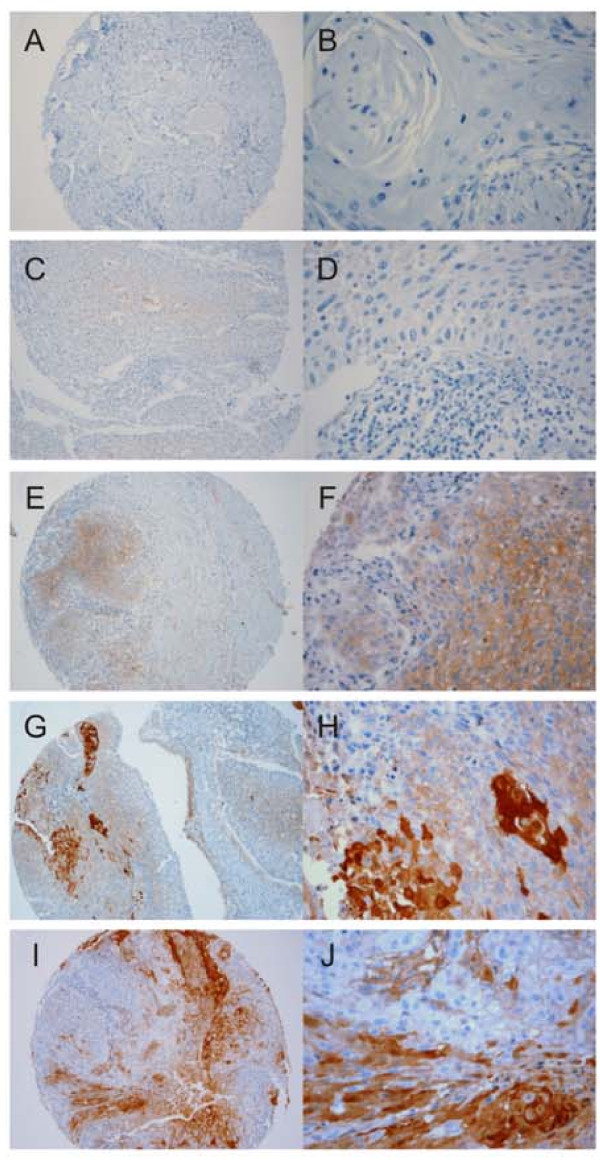
**Immunohistochemistry tissue microarray (TMA) of specimens of human larynx**. Immunostaining of the S100A7 protein using the anti-recombinant S100A7 (His-tag) rabbit serum at 1:12,000 dilution. **a **and **b **show the negative control (absence of primary antibody) for IHC. **c **and **d**, low staining (cytoplasm), spot number (417595 T2) containing squamous cell carcinoma with a 1+ staining score (same spot number). **e **and **f**, moderate staining (cytoplasm), spot number (426088 G2) containing squamous cell carcinoma. **g **and **h**, strong staining (cytoplasmic and nuclear localization); spot number (426088 T2) containing squamous cell. **i **and **j**, strong staining (cytoplasmic and nuclear localization); spot number (417739 T2) containing squamous cell carcinoma. The panels with the letters A, C, E, G and were magnified ×100, and the panels with the letters B, D, F, H and J were magnified ×400. The brownish red region indicates the immunoreactivity of the rabbit polyclonal antibody with the human S100A7 protein expressed in the tissue of the larynx with neoplasia, after being labeled with a chromogenic substrate (DAB) using the NovoLink Polymer Detection System kit (Novocastra, Newcastle Upon Tyne, UK).

The tissues spotted on the TMA slide were immunostained with the anti-recombinant S100A7 (His-tag) rabbit serum. Both neoplastic and non-neoplastic squamous epithelium reacted with the polyclonal and commercial monoclonal S100A7 antibody. Human S100A7 protein was detected by the anti-recombinant S100A7 (His-tag) rabbit serum (Figure 4).

The images of immunohistochemical reactions for the target protein shown in Figure 4 illustrate the immunoreactivity of the polyclonal antibody: Figure 4a and 4b show the negative controls (non-immune serum or absence of secondary antibody). The anti-recombinant S100A7 (His-tag) rabbit serum was able to detect both neoplastic and non-neoplastic squamous epithelium at 1:12,000 dilution (Figure 4c-j). The staining score was 1+ to 4+ with exclusive cytoplasm staining (C-F) and both cytoplasm and nuclear staining (Figure 4g-j).

We have not systematically compared the protein expression of S100A7 between normal and neoplastic tissues or with the clinical and pathological characteristics in detail. However, a preliminary analysis of the results permitted us to determine variations in the levels and location of the protein in neoplastic epithelia obtained from HNSCC (Figure 4, panels g-j). The human S100A7 protein is produced in oral tissues, with cytoplasmic localization, during the early stages of the disease and its expression increases with disease progression (cytoplasmic and nuclear localization) (Figure 4, panels g-j), suggesting its association with the progression and recurrence of HNSCC [8].

## Conclusions

The polyclonal anti-rS100A7 antibody described here provided a good signal-to-noise contrast and is suitable for immunohistochemical detection of S100A7 protein. However its reactivity with non-neoplastic squamous epithelium precludes its use as a specific marker of neoplastic tissue. It may prove to be useful as a marker of prognosis. Its potential use for other epithelial lesions besides human larynx squamous cell carcinoma and non-neoplastic larynx should be explored in further studies.

## Methods

### Cloning the S100A7 gene

The open reading frame of the human S100A7 gene was obtained by PCR using a cDNA template from tracheal tumor tissue ("Projeto Genoma Clínico", FAPESP and Ludwig Institute for Cancer Research, Brazil) [13,14]. Primers containing cleavage sites for the restriction enzyme *Xho*I (5' ATGGATCCCTCGAGATGAGCAACACTCAAG 3') and *Hind*III (5' ATAAGCTTTCACTGGCTGCCCCCGGAA 3') were synthesized by Invitrogen (Carlsbad, CA, USA). For PCR, 10 μg from trachea tissue furnished by the human tissue cDNA bank of the Regional Center for Hemotherapy of Ribeirão Preto, University of São Paulo, was used. The reaction mixture contained 1 mM dNTPs, 7.5 mM sense and complementary primers, 1 U Platinum *Taq *DNA polymerase in 50 mM Tris-HCl, pH 8.8, containing 500 mM KCl, 5 mM MgCl2 (Invitrogen) and 1% Tween 20 (Sigma, Saint Louis, MO, USA). PCR consisted of an initial denaturation step of 5 min at 94°C, followed by 35 cycles of 40 s at 94°C (denaturation), 1 min of annealing at 53°C (the specific annealing temperature for the S100A7 gene) and 1 min at 72°C (extension). A second nested PCR assay was then performed using 1 μl of the reaction mixture described above and annealing at 60°C. The amplified 306 bp product was purified on 1.0% (w/v) agarose gel using the Wizard SV Gel, PCR Clean-Up System kit (Promega, Madison, WI, USA) and inserted into the propagation vector pCR2.1-TOPO using the TOPO-TA cloning kit (Invitrogen) according to the manufacturer's instructions. The construct was introduced into *E. coli *DH5α by the thermal shock method described by Sambrook and Russell [15] and the recombinants were selected on solid Luria-Bertani (LB) medium containing ampicillin (100 μg/ml). Plasmid DNA was extracted with the plasmidPrep Mini Spin kit (GE Healthcare, Little Chalfont, Buckinghamshire, UK) according to the manufacturer's instructions. The structure of the construct was confirmed by nucleotide sequencing using the Thermocycle Sequencing BigDye Terminator kit (Applied Biosystems, Carlsbad, CA, USA) in an ABI Prism 377 automatic sequencer (Applied Biosystems). The S100A7 fragment was removed from the pCR2.1-TOPO vector by digestion with the restriction enzymes *Xho*I and *Hind*III and attached to the expression vector pAE, which contained the 6xHis-tag peptide in the N-terminal region [16], according to the protocol for the enzyme T4 DNA ligase (Promega). The construct was introduced into competent *E. coli *DH5α cells with the Subcloning Efficiency™ Chemically Competent *E. coli *kit (Invitrogen). The transformants were selected by PCR and plasmid DNA was extracted as described by Sambrook and Russell [15] and sequenced using the DYEnamic ET Dye Terminator Sequencing kit (GE Healthcare) with the automatic sequencer MegaBace™ 1000 (GE Healthcare). This indicated that the insertion of the 306 bp fragment corresponding to the S100A7 gene into the expression vector pAE was correct. The sequence of the fragment was identical to that deposited in GenBank as accession number 002963.

### Expression of rS100A7 (His-tag) protein

The *E. coli *BL21::DE3 host was transformed with the vector pAE+S100A7 and plated onto selective medium. A single colony was used for the inoculation of 200 ml CircleGrow liquid medium containing 100 μg/mL ampicillin into a 500 ml Erlenmeyer flask. The culture was maintained at 37°C and stirred until it reached an absorbance of approximately 0.5 at 600 nm. Isopropyl-β-D-1-thiolgalactopyranoside (IPTG) at 1 or 10 mM was added to try to increase the amount of protein produced.

### Purification of rS100A7 (His-tag) protein

*E. coli *BL21::DE3 cultivated in 500 ml of culture medium was centrifuged at 5,000 *g *for 10 min and the pellets were stored at -80°C. They were resuspended in 5 ml lysis buffer A (6 M GuHCl; 0.1 M NaH_2_PO_4_, 10 mM Tris-Cl; pH 8.0) and maintained at 4°C for one hour with shaking at 1,000 rpm. The lysate was centrifuged at 10,000 *g *for 30 min at 4°C (Eppendorf 5804 R) and 1.0 ml the supernatant was loaded onto an Ni-NTA SuperFlow affinity chromatography minicolumn equilibrated with buffer A (Qiagen, Hilden, Düsseldorf, Germany). The remainder of the lysate (4.0 ml) was processed at 1.0 ml/minicolumn. Each was centrifuged at 700 *g *for 2 min. After loading the minicolumn was washed with 600 μl buffer C (8 M urea; 0.1 M NaH_2_PO_4_, 10.0 mM Tris-Cl; pH 6.3) and centrifuged at 700 *g *for 2 min two times. The protein was eluted with 200 μl Buffer E (8 M urea; 0.1 M NaH_2_PO_4_, 10.0 mM Tris-Cl; pH 4.5) and centrifuged at 700 *g *for 2 min, three times.

The homogeneity of the recombinant S100A7 (His-tag) protein (10 μg) was evaluated by 12.5% SDS-PAGE [17]. The gel was stained with colloidal Coomassie blue G-250 (Serva, Heidelberg, Germany) according to Neuhoff et al. [18]. The amount of recombinant protein was measured with a DC Protein Assay kit (Bio-Rad) based on the method of Bradford [19] and determined by absorbance at 595 nm in a Versa-Max microplate reader (Molecular Devices, Sunnyvale, CA, USA).

### Characterization of the recombinant S100A7 (His-tag) by mass spectrometry

Recombinant S100A7 (His-tag) protein was characterized after *in situ *trypsin digestion of the SDS-PAGE gel band corresponding to 11.5 kDa. The band was cut from the gel, destained with 0.1 M NH4HCO3 in 50% acetonitrile, dehydrated with 100% acetonitrile, dried in a SpeedVac (Savant Inc., Ramsey, MN, USA), and rehydrated with 20 μl 0.1 M NH_4_HCO_3 _containing 0.5 μg trypsin (Promega). The protein was digested at 37°C for 24 h. The hydrolysate was desalted in a microtip filled with POROS 50 R2 resin (PerSeptive Biosystems, Foster City, CA, US) and analyzed with a MALDI-TOF/TOF mass spectrometer (Axima Performance, Kratos-Shimadzu Biotech, Manchester, UK) equipped with a collision chamber for collision-induced dissociation (CID), with helium as the collision gas. The desalted hydrolysate was dissolved in 10 μl matrix solution containing 5 mg/ml alpha-cyano-4-hydroxycinnamic acid in 0.1% trifluoroacetic acid and 50% acetonitrile. Two microliters of the sample-matrix solution were applied to the stainless steel MALDI plate and allowed to evaporate. The mass spectrometer was calibrated with a mixture of angiotensin II, ACTH fragment 18-39, bradykinin fragment 1-7 and the oxidized B chain of bovine insulin (Sigma). The PMF and MS/MS spectra were analyzed with Mascot software V.2.2.4 (Matrix Science, London, UK) and compared with the structure of S100A7 (His-tag) construct.

The molecular weight of the intact rS100A7 (His-tag) protein was determined with MALDI-TOF/TOF-MS operated in the linear mode after desalting with POROS 50 R2. The instrument was calibrated with insulin, cytochrome C, chymotrypsin and bovine serum albumin.

### Production of polyclonal antibody anti-rS100A7

The anti-recombinant S100A7 (His-tag) rabbit serum was obtained according to standard protocols [20]. Briefly, two adult female New Zealand white rabbits were immunized with 2.0 mg/animal of purified recombinant S100A7 (His-tag) protein emulsified in complete or incomplete Freund's adjuvant (Sigma) by subcutaneous or intramuscular injection, respectively. Before immunization, blood was obtained from each animal to prepare non-immune serum. The immunization efficiency was analyzed by Western blotting and serum samples were stored at -20°C.

### Reaction of rabbit polyclonal antibody with recombinant S100A7 (His-tag) protein under denaturing and reducing conditions

Western blotting was performed after 200 ng of purified recombinant S100A7 (His-tag) protein was submitted to 12.5% SDS-PAGE [17] and the protein was transferred from the gel to a polyvinyldene fluoride (PVDF) membrane in buffer containing 25 mM Tris, 192 mM glycine, pH 8.3, and 10% methanol. A constant voltage of 35 V and 100 mA was applied. A PVDF membrane and the Chromogenic Western Blot Immunodetection kit (Invitrogen) were used according to manufacturer's instructions. The membranes were incubated separately with the primary antibodies, i.e., the anti-recombinant S100A7 (His-tag) rabbit serum at 1:5,000 dilution, and the positive control monoclonal antibody S100A7 (Novocastra, Newcastle Upon Tyne, UK) at 1:1,000 dilution. Finally, the membranes were washed in MilliQ water and incubated with the chromogenic substrate 5-bromo-4-chloro-3-indolyl-1-phosphatase (BCIP) and nitroblue tetrazolium (NBT). After protein labeling, the membranes were washed with MilliQ water in order to stop the reactions.

### Interaction of the polyclonal antibody anti-rS100A7 with tissue microarray (TMA)

The anti-recombinant S100A7 (His-tag) rabbit serum was used for the detection of S100A7 by immunohistochemistry in tissue samples arranged in a TMA. Representative areas of tissues fixed in formalin and embedded in paraffin were selected randomly and used for the construction of a TMA block which contained up to four spots each of non-neoplastic tissue (Malpighian epithelium) and of neoplastic tissue (squamous cell carcinoma) from eight cases of neoplasias of the head and neck region (larynx). The TMA experiment was conducted using the polyclonal antibody at 1:2,000 to 1:16,000 dilutions. The anti-S100A7 monoclonal antibody (Novocastra) was used as a positive control at a dilution of 1:50 to 1:200. All dilutions were incubated at 4°C for 16 h after antigen recovery under the following conditions: incubation with 10 mM sodium citrate, pH 6.0, or incubation with 1 mM EDTA, pH 8.0, in a pressure cooker for 40 min, or in the absence of antigen recovery. The reaction was amplified using the NovoLink Polymer Detection System kit (Novocastra) according to manufacturer's instructions. The reaction was developed with the chromogenic substrate containing 0.10% diamine benzidine (Sigma, Saint Louis, MO, US), 0.06% hydrogen peroxide, and 1% dimethyl sulfoxide (Labsynth) in PBS buffer. Sections containing TMA were then counterstained with Harris hematoxylin and rapidly immersed in 0.5% ammonium hydroxide. Immunoreactivity was scored as follows: absent (no color) or positive (brownish red) present in up to 5% of the cells of interest (+1), present in > 5% and ≤ 25% of the cells of interest (+2), present in > 25% and ≤ 50% of the cells of interest (+3), and present in > 50% of the cells of interest (+4).

The controls were: positive - spots containing human lung - and the negative control was without the primary antibody.

## List of abbreviations

BCIP: 5-bromo-4-chloro-3'-indolyphosphate P-toluidine salt; CID: Collision-induced dissociation; DAB: 3, 3' Diaminobenzidine; His-tag: Hexa Histidine-tag; HNSCC: Head and neck spinocellular carcinoma; IHC: Immunohistochemistry; IPTG: Isopropyl β-D-1-thiogalactopyranoside; NBT: Nitroblue tetrazolium chloride; Ni-NTA: Nickel-nitrilotriacetic acid; PBS: Phosphate buffered saline; PCR: Polymerase chain reaction; PMF: Peptide mass fingerprinting; PVDF: Polyvinylidene fluoride; S100A7: S100 calcium-binding protein A7; SAGE: Serial analysis gene expression; SDS-PAGE: Sodium dodecyl sulfate polyacrylamide gel electrophoresis; TMA: Tissue microarray

## Competing interests

The authors declare that they have no competing interests.

## Authors' contributions

MRB, CDCA, WASJ and MAZ participated in the conception and design of the study. MRB, CDCA, AAM, MBAM, ICS, AW, and HJL participated in the data acquisition. MRB, CDCA, AML, MBAM, MDB, ICS, AW, VAFA, HJL, LJG, and MAZ were involved interpretation of the data. MRB, AML, and LJG participated in drafting and revision of the manuscript. MAZ conceived the study. WASJ, MDB, VAFA, LJG, and MAZ participated in the coordination of the experiments and methodology this study. All authors read and approved the final version of the manuscript.

## References

[B1] Halfpenny W Hain SF Biassoni L Maisey MW Sherman JA McGurk M FDG-PET. A possible prognostic factor in head and neck cancer Br J Cancer 2002 86 512 516 10.1038/sj.bjc.6600114 11870529 PMC2375291

[B2] Reid BC Alberg AJ Klassen AC Rozier RG Garcia I Winn DM Samet JM A comparison of three comorbidity indexes in a head and neck cancer population Oral Oncol 2002 38 187 194 10.1016/S1368-8375(01)00044-6 11854067

[B3] Hanahan D Weinberg RA The hallmarks of cancer Cell 2000 100 57 70 10.1016/S0092-8674(00)81683-9 10647931

[B4] Silveira NJF Varuzza L Machado-Lima A Lauretto MS Pinheiro DG Rodrigues RV Severino P Nobrega FG Head and Neck Genome Project GENCAPO Silva WA Jr Pereira CAB Tajara EH Searching for molecular markers in head and neck squamous cell carcinomas (HNSCC) by statistical and bioinformatic analysis of larynx-derived SAGE Libraries BMC Med Genomics 2008 1 56 73 10.1186/1755-8794-1-56 19014460 PMC2629771

[B5] Kesting MR Sudhoff H Hasler RJ Nieberler M Pautke C Wolff KD Wagenpfeil S Al-Benna S Jacobsen F Steinstraesser L Psoriasin (S100A7) up-regulation in oral squamous cell carcinoma and its relation to clinicopathologic features Oral Oncol 2009 11 1 6 10.1016/j.oraloncology.2008.11.012 19147391

[B6] Krop I März A Carlsson H Li X Bloushtain-Qimron N Hu M Gelman R Sabel MS Schnitt S Ramaswamy S Kleer CG Enerbäck C Polyak K A putative role for psoriasin in breast tumor progression Cancer Res 2005 65 11326 11334 10.1158/0008-5472.CAN-05-1523 16357139

[B7] Gagnon A Kim JH Schorge JO Ye B Liu B Hasselblatt K Welch WR Bandera CA Mok SC Use of a combination of approaches to identify and validate relevant tumor-associated antigens and their corresponding autoantibodies in ovarian cancer patients Clin Cancer Res 2008 14 764 771 10.1158/1078-0432.CCR-07-0856 18245537

[B8] Tripathi SC Matta A Kaur J Grigull J Chauhan SS Thakar A Shukla NK Duggal R Gupta SD Ralhan R Siu KWM Nuclear S100A7 is associated with poor prognosis in head and neck cancer PLoS One 2010 5 119 139 10.1371/journal.pone.0011939 PMC2914786 20689826

[B9] Madsen P Rasmussen HH Leffers H Honore B Olsen K Olsen E Kiil J Walbum E Andersen AH Basse B Lauridsen JB Ratz GP Celis A Vandekerckhove J Celis JE Molecular cloning, occurrence, and expression of a novel partially secreted protein "*Psoriasin*" that is highly upregulated in psoriatic skin J Invest Dermatol 1991 97 701 712 10.1111/1523-1747.ep12484041 1940442

[B10] Hoffmann HJ Olsen E Etzerodt M Madsen P Kruse HC Kruse T Celis JE Psoriasin binds calcium and is upregulated by calcium to levels that resemble those in normal skin J Invest Dermatol 1994 103 370 375 10.1111/1523-1747.ep12395202 8077703

[B11] Donato R Intracellular and extracellular roles of S100 proteins Microsc Res Tech 2003 60 540 541 10.1002/jemt.10296 12645002

[B12] Watson PH Leygue ER Murphy LC Molecules in focus psoriasin (S100A7) Int J Biochem Cell Biol 1998 30 567 571 10.1016/S1357-2725(97)00066-6 9693957

[B13] Brentani H Caballero OL Camargo AA Silva AM The generation and utilization of a cancer-oriented representation of the human transcriptome by using expressed sequence tags Proc Natl Acad Sci 2003 100 13418 13423 10.1073/pnas.1233632100 14593198 PMC263829

[B14] Camargo AA Samaia HPB Dias-Neto E Simão DF The contribution of 700,000 orf sequence tags to the definition of the human transcriptome Proc Natl Acad Sci 2001 98 12103 12108 10.1073/pnas.201182798 11593022 PMC59775

[B15] Sambrook J Russell DW Molecular Cloning: A Laboratory Manual 2001 3 New York: Cold Spring Harbor Laboratory Press

[B16] Ramos CRR Abreu PAE Nascimento ALTO Ho PL A high-copy T7 *Escherichia coli *expression vector for the production of recombinant proteins with a minimal N-terminal His-tagged fusion peptide Braz J Med Biol Res 2004 37 1103 1109 15273812 10.1590/s0100-879x2004000800001

[B17] Laemmli UK Cleavage of structural proteins during the assembly of the head of bacteriophage T4 Nature 1970 227 682 685 10.1038/227680a0 5432063

[B18] Neuhoff V Arold R Taube P Ehrhardt W Improved staining of proteins in polyacrylamide gels including isoelectric focusing using gels with clear background at nanogram sensitivity using Coomassie Brilliant Blue G-250 and R-250 Electrophoresis 1988 9 255 262 10.1002/elps.1150090603 2466658

[B19] Bradford MM A rapid and sensitive method for the quantitation of microgram quantities of protein utilizing the principle of protein-dye binding Anal Biochem 1976 72 248 254 10.1016/0003-2697(76)90527-3 942051

[B20] Harlow E Lane D Antibodies: A Laboratory Manual 1988 Cold Spring Harbor, NY. Cold Spring Harbor Laboratoty

